# Knowledge, attitudes and practices (KAP) towards rabies and free-roaming dogs (FRD) in Shirsuphal village in western India: A community based cross-sectional study

**DOI:** 10.1371/journal.pntd.0007120

**Published:** 2019-01-25

**Authors:** Harish Kumar Tiwari, Mark O’Dea, Ian Duncan Robertson, Abi Tamim Vanak

**Affiliations:** 1 College of Veterinary Medicine, School of Veterinary and Life Sciences, Murdoch University, Perth, Western Australia, Australia; 2 Ashoka Trust for Research on Ecology and Environment (ATREE), Bangalore, India; 3 Ausvet, Fremantle, Western Australia, Australia; 4 Wellcome Trust/DBT India-Alliance Fellow, Hyderabad, India; 5 China-Australia Joint Research and Training Center for Veterinary Epidemiology, Huazhong Agricultural University, Wuhan, Hubei, China; 6 School of Life Sciences, University of KwaZulu-Natal, Durban, South Africa; National Institutes of Health, UNITED STATES

## Abstract

The lack of awareness about dog-bite related rabies in the rural population of developing countries, including India, is a major impediment to controlling the incidence of disease in humans. A survey of 127 rural residents was undertaken in Shirsuphal village in western India using a structured questionnaire to assess the influence of demographic and pet/livestock owning characteristics on the knowledge, attitudes and practices of the respondents towards rabies and free roaming dogs (FRD). Multivariable logistic regression models were constructed and the knowledge of the rural residents of Shirsuphal village was found to be significantly influenced by family size (OR 2.1, 95%CI 1.0–4.6, p = 0.04) and poultry ownership (OR 2.3, 95%CI 1.1–4.9, p = 0.03), while their attitudes towards FRD was significantly influenced by age of the respondents (OR 2.6, 95% CI 1.2–5.8) and ownership of cattle/buffalo (OR 2.2, 95% CI 1.1–5.5). Although the knowledge score about rabies was high, a comprehensive understanding of the disease was lacking. Concerted efforts to widen the knowledge about rabies and promote healthier practices towards FRD are recommended.

## Author summary

The rural population in developing countries, such as India, are most vulnerable to transmission of rabies, especially due to animal-bites. The widespread trust in traditional healing practices for treating animal-bite injuries undermines the importance of seeking post-bite vaccination against rabies. In spite of a wider acknowledgement of the role of free-roaming dogs in the transmission of rabies virus in rural human populations, the latter’s attitudes towards this vector host remains influenced by the social, cultural and religious beliefs prevalent in the area. This study explores the awareness level and perception of villagers not only towards rabies but also the free-roaming dogs in the locality. Although the rural participants surveyed in this study were aware of the disease, gaps were revealed in the comprehensive knowledge about rabies and its transmission. The villagers were also found lacking in adequate practices towards free-roaming dogs that can remarkably reduce the incidence of the disease. Concerted efforts to widen the knowledge about rabies and promote healthier practices towards free-roaming dogs is recommended.

## Introduction

India has the world’s highest number of dog-bite related rabies deaths, most of whom are people of low socio-economic background from rural areas [1, 2]. A gross lack of awareness about rabies in rural India is one of the factors that leads to high human mortality from the disease [3]. Although mortality can be prevented through prompt washing of bite wounds with soap and water [4, 5], along with timely administration of rabies immunoglobulins (RIG) and anti-rabies vaccines (ARV) [6, 7], these practices are potentially undermined by widespread traditional healing practices, such as application of chilli/turmeric powder to bite wounds [8, 9]. Policy makers and the general population lack awareness about the impact of rabies [10, 11] which results in insufficient vaccination coverage of dogs, poor knowledge of post-exposure prophylaxis (PEP) amongst medical professionals and unreliable supply of ARV and RIG [12]. Also, insufficient financial resources, poor health care infrastructure and inadequate reporting systems leads to an underestimation of the true public health impact of rabies in India [10, 13].

Free-roaming dogs (FRD), which are responsible for 96% of all human rabies deaths in India, are ubiquitous in both rural and urban localities/communities [2]. Management of the FRD population, along with responsible ownership of dogs, are key strategies to minimise human deaths from dog-bite related rabies [14]. Although studies in India have assessed the knowledge, attitudes and practices (KAP) of communities towards rabies [15–18], studies on the community’s attitudes and understanding of FRD are lacking.

Although India contributes 4.4% of the total global research output on rabies, there is a lack of studies focussing on the vector demography, risk factors, epidemiological studies and economic evaluations of the disease [19]. There is also a lack of awareness by the rural population about rabies control programmes [20]. Paucity of activities that can transfer knowledge about the disease to the rural population is a key concern for policy makers [21] and the importance of epidemiological studies to assess the awareness level and practices of people regarding aspects of rabies control is paramount in this context [12, 20, 22]. While there have been a number of hospital based studies that have assessed KAP about rabies involving dog-bite victims, community based studies are virtually lacking in India [23]. In view of this deficiency, a cross-sectional community study was designed in rural Baramati, western India, in the village of Shirsuphal to assess the: (1) KAP of the rural community towards rabies; (2) KAP of the rural community towards FRD population management; and (3) KAP of rural dog owners on responsible ownership of dogs.

## Materials and methods

### Sample size

Recent surveys conducted in rural areas in India near to the present location formed the basis for calculating the sample size for this study. A weighted measure (93.6%) of respondents having heard of rabies from four community based cross-sectional studies carried out in neighbouring states was used to calculate the target sample size with 95% confidence and 5% error rate [5, 16, 18, 24]. With 1161 households in Shirsuphal, the required sample size was estimated to be 86. However, we had sufficient resources to administer the questionnaire to 132 respondents, of which five failed to complete the survey. Consequently the responses of 127 participants were included in the survey analysis, thus achieving a confidence level of 98% at 2% error.

### Study area and survey procedure

A cross-sectional household survey was undertaken in the Shirsuphal village of Baramati Town of Pune District in Maharashtra state in western India from 13^th^– 21^st^ June 2016. The village has a population of 5512 in 1161 households (www.censusindia.gov.in, accessed on 08 October 2015). The majority of the villagers are farmers, although there are some professionals and small business owners. Some farmers have also taken up poultry farming in recent years. No rabies awareness campaign or dog population control measures had been conducted in the area prior to this survey.

The houses are divided into four clusters of a similar population size in the village, however they are not numbered. Although the total number of households in each cluster was known, there was limited information available regarding the number of households in each lane, consequently a door-to-door survey method was followed using a rolling sample method where the first randomly selected household provided information about the next available household within the cluster [25, 26]. To avoid potential bias being introduced by the respondents nominating relatives or friends, they were requested to nominate a household in a different direction to that of their friends and relatives within the cluster. A total of 33 households from each cluster were included for the questionnaire survey. The household head was approached to complete the questionnaire and if he/she were not available or not willing then a household member who was older than 18 years of age was invited to complete the questionnaire. A document outlining informed consent was read out to them in the local language (Marathi) and verbal consent obtained before administering the questionnaire. In the event of the household declining to participate in the survey then the adjacent house was selected for inclusion in the study.

### Questionnaire design

The KAP survey was designed to: identify gaps in awareness about rabies; assess the practices that potentially contributed to the persistence of the disease in the village; assess the attitudes of the community towards FRD; and assess the attitudes of dog owners towards their pets. The questionnaire consisted of closed questions on: (1) household information to assess the socio-economic status and resident profile (age, education, occupation, religion, family size, number of children below 14 years of age, and pet and livestock ownership); (2) knowledge, attitudes and practices regarding rabies (a total of 16 questions—11 pertaining to knowledge and five pertaining to attitudes and practices towards rabies, respectively); (3) attitudes and practices towards FRD (seven questions); and (4) pet care practices adopted by dog-owners (15 questions asked only to respondents who owned pet dogs). The questions were read out to the respondents in their local language (Marathi) by the interviewer and their answers were recorded in English (Appendix).

### Data management and analysis

Answers to the questions were tabulated in a spreadsheet (Microsoft Excel, Microsoft Corp., Redmond, WA, USA). “Not sure” responses were combined with the “No” option and “NA (not applicable)” responses were removed from the study prior to subsequent statistical analysis using the R Programming Environment [27].

A matrix was developed to categorise the respondents into high, medium and low socio-economic status on the basis of their educational qualification and occupation on a design based on www.praja.org (accessed 18 March 2016). Subsequently, the high and medium categories were merged to obtain a binomial distribution of respondents into two socio-economic divisions: low and medium/high. The age of the respondents and the family size of the households was dichotomised into two age groups based on the median age/family size (S1 File).

#### Analyses of the responses to the individual questions

Bivariate analyses were performed by using the chi-squared (χ^2^) test to compare the responses to each question relating to Sections 2 and 3 of the questionnaire. Section 2 comprised of two categories: (a) knowledge of rabies; and (b) attitudes and practices towards preventing and controlling rabies. Only dog-owners were asked the questions in Section 4. The characteristics of the owned dogs and their owners’ perceptions and practices towards FRD were analysed using test of association (odds ratio). Odds ratios were calculated using the “odds ratio” package in R [28].

#### Univariable and multivariable analyses

Three separate multivariable models were developed to investigate the association of various factors with the socio-demographic characteristics of the respondents. Initially, the cumulative score obtained for questions pertaining to the three response criteria (knowledge and awareness about rabies; attitudes and practices towards rabies; and attitudes and practices towards FRD, respectively) was converted into binomial outcomes by categorising the respondents as having scored above or below the median score for each response criteria. The association between this outcome variable and various demographic and household factors was then evaluated using a χ^2^ test or Fisher’s exact test. All explanatory variables with a p ≤ 0.25 were offered to the multivariable logistic regression models. The reduced subset models were developed using backward elimination based on the AIC (Akaike Information Criteria) score for each model. The final multivariable logistic regression models were evaluated using Pearson’s and Deviance residuals and its goodness—of-fit was assessed by the Hosmer-Lemeshow test [29]. Variables with p < 0.05 were retained in the final model.

## Results

### Demographic and socio-demographic characteristics of the respondents

The demographic and household characteristics of the respondents are presented in Table 1. The age groups and the family-size was dichotomised at the median age, i.e. 35 years (≤34 years and ≥35 years of age) and the median family size, i.e. 6 members (<6 and ≥6), respectively.

**Table 1:** Demographic characteristics of the respondents (n = 127).

Variable/Category	n (%)
**Gender**	
Male	89 (70)
Female	38 (30)
**Age(years)** (range 18–72, average 39.4)	
18–34	64 (50)
≥ 35	63 (50)
**Socio-economic status**	
High/middle	52 (41)
Low	75 (59)
**Family size** (range 2–16, average 6.2)	
<6	64 (50)
≥6	63 (50)
**Children (≤ 14 years) are present in the family**	
Yes	74 (58)
No	53 (42)
**Own a pet(s)**	
Yes	83 (65)
No	44 (35)
**Own a dog(s)**	
Yes	67 (53)
No	60 (47)
**Own livestock**	
Yes	93 (73)
No	34 (27)
**Own cattle/buffalo**	
Yes	67 (53)
No	60 (47)
**Own sheep/goats**	
Yes	76 (60)
No	51(40)
**Own poultry**	
Yes	71 (56)
No	56 (44)

### Respondent’s knowledge, attitudes and practices regarding rabies

#### Respondent’s knowledge and awareness of rabies

Most respondents (97%) had heard of rabies. Of these, 98.4% knew that rabies could be transmitted through animal bites, although less than half (50, 40.7%) were aware that it could also be transmitted through licks/scratches. All respondents knew that dogs were capable of transmitting rabies but only approximately a quarter recognised that cats (22%) could also transmit the virus. Most people (86%) knew that rabies was fatal once acquired and 80% were aware that it could be prevented. Of this latter group a similar proportion (73%) knew that it could be prevented by administering PEP to dog-bite victims or by vaccinating dogs against rabies. Respondents from smaller households (<6 members) were more aware that rabies could be prevented by vaccination (OR 2.8, 95%CI 1.2–6.7, p = 0.01) than respondents from larger households.

The association between the descriptive characteristics and the knowledge of the participants is presented at Table 2. Smaller family size (<6 members) and poultry ownership were found to influence knowledge about rabies in the final multivariable model (OR 2.1; 95%CI 1.0, 4.6; 2.3 95%CI 1.1, 4.9, respectively) (Table 3). The model was a good fit of the data with a Likelihood ratio (χ^2^) test value of 8.7 (p = 0.04) and a Hosmer—Lemeshow goodness of fit test result of 0.78 (p = 0.37).

**Table 2:** Association of the knowledge of the participants about rabies with various descriptive variables.

Variable/category (n)	Number knowledgeable (%)	P-value (χ^2^ test)	OR (95% CI)
Gender			
Female (38)	19 (50)		1
Male (89)	57 (64)	0.14*	1.8 (0.8–3.9)
Age(years)			
≤ 34 (64)	36 (56)		1
≥35 (63)	40 (63)	0.4	1.3 (0.7–2.8)
Social status			
Low (75)	43 (48)		1
High/middle (52)	32(61)	0.5	1.2 (0.6–2.7)
Family size			
<6 (64)	33 (52)		1
≥6 (63)	43 (68)	0.05*	2.0 (1.0–4.2)
Children (≤ 14 years) present in the household			
No (53)	33 (62)		1
Yes (74)	43 (58)	0.63	0.8 (0.4–1.7)
Pet ownership			
No (44)	25 (57)		1
Yes (83)	51 (61)	0.61	1.2 (0.6–2.5)
Dog ownership			
No (60)	32 (53)		1
Yes (67)	44 (66)	0.15*	1.7 (0.8–3.4)
Livestock ownership			
No (34)	19 (56)		1
Yes (93)	57 (61)	0.58	1.2 (0.5–2.8)
Cattle/buffalo ownership			
No (59)	32 (54)		1
Yes (68)	44 (65)	0.23*	1.5 (0.7–3.1)
Sheep/goat ownership			
No (50)	26 (52)		1
Yes (77)	50(65)	0.14*	1.7 (0.8–3.5)
Poultry ownership			
No (53)	26 (49)		1
Yes (74)	50 (68)	0.03*	2.1 (1.0–4.5)

*Variables offered to the multivariable model

**Table 3:** Final multivariable logistic regression model of factors associated with respondent’s knowledge of rabies.

Variable	Coefficients (β)	SE	p-value	OR (95%CI)
Constant	-0.45	0.5	-	-
Family size				
≥6				1.0
<6	0.77	0.38	0.04	2.1 (1.0–4.6)
Poultry ownership				
No				1.0
Yes	0.84	0.38	0.03	2.3 (1.1–4.9)

Likelihood ratio (χ^2^) test = 8.7; p = 0.04; Hosmer—Lemeshow goodness of fit test = 0.78; p = 0.37)

#### Respondent’s attitudes and practices towards rabies

A majority (87%) of respondents were aware of the ineffectiveness of traditional applications, such as chilli /turmeric powder. Less than half (42%) of respondents believed that washing bite wounds with soap and water was beneficial. Nearly all respondents (97%) would recommend a dog-bite victim attend a hospital. Although most (92%) respondents believed that restricting the FRD population could help control rabies, only 73% would report the presence of a rabid dog to the municipal authorities. The sheep/goat owners were more likely to perceive wound cleaning as useful (OR 2.4 95% CI 1.2–5.4, p = 0.01), while households that owned poultry were found less likely to report the presence of a rabid dog to the authorities (OR 0.3, 95%CI 0.13–0.7, p = 0.01).

The association between the various descriptive variables and the attitudes and practices of the participants towards rabies are presented in Table 4. Pet ownership (p = 0.11), dog ownership (p = 0.1), and poultry ownership (p = 0.19) were offered to the multivariable logistic regression model. A stable multivariate logistic regression model with significant p-values could not be generated.

**Table 4:** Association of the attitudes and practices of the respondent’s about rabies with various descriptive variables.

Variable/category	Respondents with positive attitudes and practices (%)	P-value (χ^2^test)	OR (95% CI)
Gender			
Female (38)	30 (79)		1
Male (89)	63 (71)	0.38	0.6 (0.2–1.5)
Age(years)			
18–34 (64)	49 (77)		1
≥35 (63)	44 (70)	0.39	0.71 (0.32–1.57)
Social status			
Low (75)	54 (73)		1
High/middle (52)	39 (75)	0.7	1.16 (0.52–2.66)
Family size			
<6 (64)	46 (72)		1
≥6 (63)	47 (75)	0.74	1.14 (0.5–2.5)
Presence of children (≤ 14 years) in the household			
No (53)	38 (72)		1
Yes (74)	55 (74)	0.74	1.14 (0.5–2.5)
Pet ownership			
No (44)	36 (82)		1
Yes (83)	57 (67)	0.11*	0.49 (0.17–1.2)
Dog ownership			
No (60)	48 (80)		1
Yes (67)	45 (67)	0.1*	0.5 (0.22–1.15)
Livestock ownership			
No (34)	24 (71)		1
Yes (93)	69 (74)	0.68	1.2 (0.48–2.8)
Cattle/buffalo ownership			
No (59)	43 (73)		1
Yes (68)	50 (73)	0.93	1.03 (0.46–2.3)
Sheep/goat ownership			
No (50)	36 (72)		1
Yes (77)	57 (74)	0.8	1.1 (0.48–2.4)
Poultry ownership			
No (53)	42 (79)		1
Yes (74)	51 (69)	0.19*	0.5 (0.24–1.3)

*Variables offered to the multivariable model

### Respondent’s attitudes and practices towards free-roaming dogs (FRD)

The responses of the participants to the questions pertaining to attitudes towards FRD are presented in Table 5. The younger respondents (≤34 years) did not consider FRD a threat to human health (OR 0.2, 95% CI 0.04–0.97, p = 0.05), and were more likely to feed them (OR 2.2, 95%CI 1.1–4.5, p = 0.04) than older participants. Participants from the high/middle socio-economic level considered FRD were useful (OR 3.09, 95%CI 1.06–8.97, p = 0.03), were likely to feed them (OR2.81, 95%CI 1.34–5.88, p = 0.005), and would take an injured stray dog to a veterinarian (OR 2.33 95%CI 1.0–5.48, p = 0.04). The respondents from the low socio-economic level believed that the responsibility of the health and vaccination of FRD was with the households that fed/sheltered them (OR 2.3, 95%CI 1.04–5.1, p = 0.03), a perception similar to dog owners (OR 2.9, 95%CI 1.3–6.6, p = 0.04). A significant number of poultry owners reported that FRD attacked their backyard poultry for food (OR 3.2, 95%CI 1.07–12.1, p = 0.02). The association between the various descriptive variables and the attitudes and practices of the participants towards FRD is presented in Table 6. The respondent’s age and ownership of cattle/buffalo (OR 2.6, 95%CI 1.2–5.8; OR 2.2, 95%CI 1.1–5.5, respectively) had a positive influence on their attitudes towards FRD in the final multivariable logistic regression model (Table 7). The model was a good fit of the data with a Likelihood ratio (χ^2^) test value of 10.33 (p = 0.006) and a Hosmer—Lemeshow goodness of fit test result of 0.008 (p = 0.927).

**Table 5:** Responses to various questions pertaining to attitudes and practices regarding free- roaming dogs.

Criteria		n (%)
Are there FRDs in your locality?		127 (100)
**What are the source of FRD?**		
Breeding of local FRDs		99 (78)
Nearby villages		16 (13)
Pets abandoned by villagers		11 (9)
**Are FRD useful for the society? (Yes)**		18 (14)
For guarding premises		4 (23)
Keep away wild animals		3 (18)
Keep away thieves		11 (71)
Are FRD a nuisance to the society? (Yes)		109 (86)
Are FRD a threat to human health? (Yes)		116 (91)
**Where do the FRD get their food?**		
Garbage dumps		79 (62)
Meat shop/Poultry farm waste		49 (39)
Fed by residents		12 (9)
**Do you ever feed a FRD? (Yes)**		50 (39)
**Reasons for feeding FRD**		
Religious reasons		41 (84)
Compassion		45 (90)
Better than wasting the left-over food		42 (84)
**How would you rank health of FRD in your locality?**		
Good		51 (40)
Average		44 (35)
Poor		32 (25)
**Would take an injured FRD to a veterinarian? (Yes)**		28 (22)
Should residents who feed/shelter FRDs be responsible for their health/vaccination?	Yes	34 (27)
	No	93 (73)
Is health/vaccination of the FRDs a responsibility of the government?	Yes	119 (94)
	No	8 (6)
**In your opinion which is the best way to control the FRD population?**		
Culling		16 (13)
Impounding		43 (34)
ABC		52 (41)
Garbage management		13 (10)
Not sure/others		3 (2)

**Table 6:** Association of the attitudes and practices of the community regarding free-roaming dogs with various descriptive variables.

Variable/category	Respondents with positive attitudes towards free-roaming dogs (%)	P-value (χ^2^test)	OR (95% CI)
Gender of participant			
Female (38)	27 (71)		1
Male (89)	58 (65)	0.52	0.77 (0.32–1.73)
Age(years) of participant			
≤ 34 (64)	37 (58)		1
≥35 (63)	48 (76)	0.03*	2.3 (1.08–5.07)
Social status of household			
Low (75)	56 (75)		1
High/middle (52)	29 (56)	0.02*	0.43 (0.2–0.91)
Family size			
< 6 (64)	42 (66)		1
≥6 (63)	43 (68)	0.75	1.12 (0.53–2.38)
Children (≤ 14 years) present in household			
No (53)	35 (66)		1
Yes (74)	50 (68)	0.85	1.07 (0.5–2.27)
Pet ownership			
No (44)	27 (61)		1
Yes (83)	58 (70)	0.33	1.4 (0.67–3.15)
Dog ownership			
No (60)	40 (67)		1
Yes (67)	45 (67)	0.75	1.02 (0.48–2.15)
Livestock ownership			
No (34)	20 (59)		1
Yes (93)	65 (70)	0.24*	1.6 (0.7–3.6)
Cattle/buffalo ownership			
No (59)	34 (58)		1
Yes (68)	51 (75)	0.04*	2.2 (1.0–4.7)
Sheep/goat ownership			
No (50)	31 (62)		1
Yes (77)	54 (70)	0.34	1.4 (0.67–3.06)
Poultry ownership			
No (53)	34 (64)		1
Yes (74)	51 (69)	0.57	1.2 (0.58–2.62)

*Variables included for building the multivariable model

**Table 7:** Final multivariable logistic regression model of factors associated with respondent’s attitudes and practices towards free-roaming dogs.

Variable/category	Coefficients (β)	SE	P-value	OR(95% CI)
Constant	-0.2	0.34		
Age(years)				
≤ 34				1
≥35	0.96	0.4	0.02	2.6 (1.2–5.8)
Cattle/buffalo ownership				
No				1
Yes	0.91	0.4	0.02	2.2 (1.1–5.5)

Likelihood ratio (χ^2^) test = 10.33; p = 0.006; Hosmer-Lemeshow goodness of fit test = 0.008; p = 0.9

### Characteristics of dog owners

The characteristics of the dog owners in this study are presented in Table 8. Dog-owners who had a negative perception of FRD were less likely to seek veterinary attention (OR 0.3, 95%CI 0.1–1, p = 0.047) for their pets. Dog owners who adopted their pets “off the street” were less likely to get their dogs vaccinated than those who either purchased them or were given them (OR 0.08, 95%CI 0.01–0.4, p = 0.001). Dog owners who had an adequate knowledge about rabies (76, 59.8%) or possessed a perception that controlling FRD would help control rabies (85, 66.9%) were not significantly different from those who didn’t own dogs (p = 0.29, p = 0.75).

**Table 8:** The characteristics of the owned dogs and owner’s perceptions and practices about their pets.

Criteria	Number of respondents (%)
**Breed of dog owned**	
Local	56 (84)
Pedigree	8* (12)
Mixed	3** (4)
**Source of dog owned**	
Adopted	46 (69)
Gifted	11 (16)
Purchased	6 (9)
Offspring of owned bitches	4 (6)
**Preference for pedigreed breeds**	25 (37)
**Reasons for preferring a pedigreed dog**	
Intelligence	17 (40)
Cleanliness	3 (7)
Social status	3 (7)
No specific reasons	2 (5)
**Are the pet dog(s) confined and restricted?**	
Yes	56 (84)
No	11 (16)
**Is your dog(s) supervised when not restricted?**	
Respondents saying ‘always’	26 (46)
Respondents saying ‘sometimes’	12 (22)
Respondents saying ‘rarely’	5 (9)
Respondents saying ‘never’	13 (23)
**Have you ever visited a veterinarian**	19 (28)
**Has your dog ever been vaccinated against rabies**	9 (12)
**Is your dog(s) sterilised?**	4 (6)
**If not sterilised why it hasn’t been sterilised?**	
Unaware of the procedure	15 (24)
Unavailability of service	9 (14)
Consider it a cruel practice	5 (8)
Pet reared for breeding	5 (8)
Cost of the procedure	1 (1.5)
Reduces aggressive nature of the guard dogs	1 (1.5)
Too young for procedure	20 (32)
No specific reason	7 (11)

*5 Labradors, 2 Pomeranians and 1 Pug

**Cross between pedigreed and FRD

## Discussion

This questionnaire study was undertaken to assess the KAP of a rural community in Shirsuphal village to better understand the challenges facing rural India in the quest to reduce the incidence of dog-bite rabies. Although many studies have identified a lack of awareness about rabies in the rural, socio-economically poor communities of India [2, 21, 30, 31], this is one of the first studies that relates the KAP of a rural community towards rabies with FRD.

The proportion of respondents who had heard about rabies (96.4%) was higher in this survey than in previous studies from South India and from in and around Delhi [8, 17, 32], although it was similar to studies conducted close to the present location in rural areas of Pune, Gujarat, Karnataka and Tamil Nadu [5, 16, 18, 24]. This increase in awareness about rabies could be attributed to prioritising the disease as important by the Government of India in the 11^th^ five-year-plan (2007–2012) which included creating awareness of the disease as a key focus [33]. There is also an improved availability of PEP at local Public Health centres (personal communication, Medical officer at Public health centre at Shirsuphal) which could also have contributed to this improved awareness. In spite of the increased proportion of respondents having heard about the disease, comprehensive understanding about the disease was lacking amongst most participants. Although the majority of the respondents were aware that rabies: could be transmitted through dog-bites, is fatal once clinical signs develop and can be prevented through post-bite anti-rabies vaccination or prophylactic vaccination of dogs, most were unaware it could be transmitted through licks/scratches from a rabid animal or through rabid cats. Furthermore the fact that 29% of the participants were not aware of PEP or prophylactic vaccines is of concern, particularly if they or a family member is bitten by a rabid dog. As no rabies awareness campaign had ever been delivered in the village prior to the current study, this study highlights the need to expand existing programmes and to develop a structured awareness campaign in this and potentially other locations.

In this study respondents from families of a smaller size (<6) were more knowledgeable about rabies (OR 2.15, p = 0.04), which is likely associated with the make-up of the families as they largely comprised adults older than 35 years of age (OR 2.08, p = 0.04) who had a higher overall knowledge on rabies. This would most likely arise from more opportunity to have read, seen or heard about the disease and how it can be controlled through the media or through discussions with other community members than younger individuals. This finding is consistent with another recent KAP study in south western Ethiopia that showed that older age groups were more knowledgeable about rabies than younger age groups [34] but was contrary to the findings of Herbert, Basha (17) where older age groups had an overall lower literacy than younger age groups. However in the latter survey the participants were “slum dwellers” who are likely to be less literate than the participants of this survey in Shirsuphal village.

Rabies causes economic losses in rural communities through deaths of livestock and poultry [35, 36]. Although there have been no studies conducted in India to evaluate livestock losses from rabies in India, dog-bites have been reported in almost all domesticated species [37]. In the current study, 20% of the participants reported that packs of FRD often attacked backyard poultry for food. This is probably linked with the fact that poultry owners were more likely to consider FRD a nuisance and consequentially they may have a better understanding of the diseases transmitted by them.

Unexpectedly more people with a good overall understanding of the practices to adopt to control rabies did not consider wound cleaning beneficial (OR 3.6, p = 0.009) as reported in other studies also undertaken in India [38–40]. This finding highlights a lack of understanding about important measures to prevent infection. There is a Public Health Centre (PHC) within the village and PEP is available (information obtained from the Village head and PHC staff), but a lack of awareness by the general community about preventive practices hinders seeking prompt medical attention [41, 42]. A concerted effort to improve the knowledge of rural communities is paramount to raising the awareness of practices to adopt to counter rabies.

The current study found that the majority (86%) of participants felt that FRD were a nuisance and a similar proportion (91%) considered that they were a threat to human health. Similar perceptions have been reported from the neighbouring country of Bhutan [43, page 97]. Garbage sites and waste from meat/poultry shops were identified as the main sources of food for the FRD, as also reported by El Berbri, Ducrotoy [44] in Morocco. Effective management of garbage can help reduce the nuisance arising from FRDs and the incidence of dog bites [45, 46]. Animal Birth Control or sterilisation programs were the most favoured means for controlling the dog population, which was not unexpected as non-government organisations (NGO) and Government agencies have promoted ABC programs over the last few years [39, 47, 48], even though none had been conducted in the village.

Respondents from a low socio-economic level were less likely to consider FRD useful, feed them or take an injured dog to a veterinarian than participants from high/middle levels. Conversely, respondents from the high/middle socio-economic levels had more sympathetic and positive attitudes towards FRD. This could be exploited for the promotion of responsible dog ownership, including adopting FRD [14]. This is also corroborated by our finding that respondents from high/middle socio-economic class (OR = 2.9, p = 0.02), and those from smaller families (OR = 2.1, p = 0.07) were more likely to take an injured dog to a veterinarian. The smaller families in Shirsuphal usually comprised adults and elderly people who may be more compassionate with stronger religious beliefs on the importance of feeding and/or caring for dogs than younger members of larger families. Not surprising, respondents from the high/middle socio-economic class were also more likely to feed a FRD (OR = 2.8) as a result of potentially having more disposable income than those from a lower class. Most respondents believed that it was the government’s responsibility to mass vaccinate FRD. The cost of the rabies vaccine and the difficulty in administering prophylactic vaccination to dogs is a plausible explanation why people of a low socio-economic status from rural areas are unable or unwilling to provide these services [49, 50].

Households with children were more likely to consider FRD a threat to human health, most likely arising from the higher incidence of dog-bites in children reported in developing countries [3, 23]. Livestock owners were less likely to feed a stray dog (OR 0.39, p = 0.02) than non-livestock owners and this perception may arise from the direct economic impact of injuries/deaths or dog-bite rabies in livestock as a result of dog attacks from FRDs.

Some unexpected findings also arose from the current survey. For example, although only 9% of respondents believed that feed provided by villagers was a major source of food for FRD, a larger proportion (39%) actually admitted to feeding them. Similarly, the respondents who fed them surprisingly had a lower acceptance for FRD (OR 0.08). It is likely that feeding stray dogs is strongly influenced by religious convictions, as most of the respondents were followers of Hinduism, a religion which is tolerant of animals and caring for them [51, 52], but, apparently it did not motivate them sufficiently to accept responsibility for immunisations, probably also due to issues of availability of vaccines and their cost.

The “age of the respondents” and “cattle/buffalo ownership” significantly influenced the perception of the respondents towards FRD as older respondents (> 35 years of age) had a better understanding of the problems posed by these dogs (OR 2.6, p = 0.016), which is probably linked with their corresponding more awareness of rabies. Cattle/buffalo owners were found to have an overall positive attitude (OR 2.18, p = 0.04) towards FRD. Cattle/buffalo owners were more likely to own a dog (OR 4.3, p <0.001) than non-cattle/buffalo owners, which may justify their positive attitudes towards practices that help manage the FRD population.

Most dog owners (72%) had adopted offspring of FRD, indicating that the rural population is not averse to adopting local breeds. Similar results were reported in a KAP study in Nepal where 65% of pet dogs were the progeny of street dogs [53]. Responsible ownership is an essential tool for population management of dogs and rabies awareness campaigns should incorporate aspects of responsible dog ownership to reduce the number of FRD [14]. Although 84% of dog owners said that they confined their dog(s), 61% also said that there were occasions when their pets wandered freely unsupervised. This would increase the risk of owned dogs contracting rabies. This is a significant health risk particularly as only 13% of dog owners had their dogs immunised against rabies. Not surprisingly dog owners with a positive perception on rabies and FRD were more likely to get their dogs vaccinated and sterilised, demonstrating the importance of educational campaigns in disease control and population management.

As rabies is an ancient disease, and India has the largest population of FRD in the world, it is not surprising that most participants had heard of the disease. However even though one has heard about a disease, one’s knowledge about the practices that can truly reduce the incidence may be minimal. We suggest that instead of determining the sample size based upon “have you heard about the disease”, it may be more appropriate to base it on “can rabies be cured?” or “can rabies be prevented?” to obtain more robust results of the level of knowledge and practices to reduce infection in humans and other animals. Another limitation of this study was that only adults (> 18 years) were interviewed. Understanding the knowledge level of children would be useful information as targeted education of this group can result in long-term community changes [54]. This is even more important for rabies because younger people are more likely to interact with FRD and be at greater risk of contracting the disease. As a household was represented by only one respondent, we also accept that the opinion of one person may not indeed be a true representation of overall knowledge, attitudes and practices of all members in a family. Finally, none of the variables analysed in this study were found to significantly influence the practices considered favourable for the elimination of rabies. This could mean that variables/factors other than those included in the current questionnaire study were associated with the villagers’ attitudes on practices regarding rabies. Potentially, religion may play a role; however in this study the majority of participants were Hindus, precluding a comparison between different religions. Expanding the size of the study may help overcome this limitation. This study analysed data using binary logistic regression analyses which required the KAP scores of the respondents to be dichotomised at the median resulting in inclusion or exclusion of some respondents with scores near to the median. We believe that with such categorisation some factors with significant difference in scores might have been missed. Also, it may have given rise to type 1 errors such as the high knowledge scores observed for poultry owners. We also analysed the data using Poisson regression (results not shown) and found that no factors were significant on the univariable analyses. However, it is possible that this may not be the case with a larger sample size and hence we suggest that alternative regression modelling techniques may be considered in future studies to overcome this limitation.

The fight against rabies in India is unlikely to be successful unless there is concerted effort to improve the knowledge of the community about the disease. Most villagers believe that only dogs can transmit rabies; however the dangers for the rural population from cattle, buffalo, sheep or goats which contract rabies from dog-bites can still be significant [55]. The usefulness of thorough cleaning of dog-bites was not known by the majority of respondents highlighting the need to emphasise this basic, yet important fact. A structured and sustained information campaign to raise the level of awareness and improve practices against rabies is an important tool which requires serious consideration, especially in rural areas. Focussed campaigns to advise healthy practices to adopt to care for a pet are recommended for dog owners. The villagers were receptive to the conducting of mass vaccination and sterilisation campaigns and this sentiment should be utilised to immunise FRD and control their numbers in the future, although sourcing sufficient funds for on-going programs could be challenging. A major finding of this study was the positive attitudes of residents from the high/middle socio-economic level towards FRD, which could be used to encourage adoption of dogs and responsible dog ownership. In spite of the study limitations, this study highlighted that people lack comprehensive knowledge about rabies and their perception of FRD is influenced by incomplete or incorrect information. Further research is recommended to analyse the effect of predictors, such as distance from PHCs or willingness to pay for vaccination of dogs. It is also recommended to explore the use (utility) of FRD which will positively influence peoples’ attitudes towards indigenous FRD on Indian streets. Interventions for rabies control should be seen as a key component of dog health and population management.

### Ethical approval

This study involved survey of rural residents of Shirsuphal village in Baramati town of Pune district, Maharashtra, India and the ethics approval was obtained from the Murdoch University Human Ethics Committee (permission number: 20/2016). Oral consent was obtained from all respondents prior to their participation (Appendix).

## Supporting information

S1 File Table S1-S4. (DOCX)

S2 File Appendix rural KAP (Ethics approval, questionnaire—English & Marathi, oral consent form—English and Marathi). (PDF)
