# Supplementary material for: Knowledge, attitudes and practices (KAP) towards rabies and free-roaming dogs (FRD) in Shirsuphal village in western India: A community based cross-sectional study
Source: PLoS Negl Trop Dis. 2019 Jan 25;13(1):e0007120. doi: 10.1371/journal.pntd.0007120 (PMC6364945; doi:10.1371/journal.pntd.0007120)
Supplement: S1 File — (DOCX) [file pntd.0007120.s001.docx]

| Education level Occupation/Trade | No Formal  Education | PRIMARY | SECONDARY | MATRICULATE | COLLEGE | GRADUATE | POST -GRADUATE |
| --- | --- | --- | --- | --- | --- | --- | --- |
| UNSKILLED | F | F | F | E | E | E | D |
| SKILLED | F | F | E | E | E | D | D |
| SMALL TRADING | F | E | E | E | D | D | D |
| SHOP OWNER | E | E | E | D | D | D | C |
| BUSINESS | E | E | D | D | D | C | C |
| CLERICAL | E | D | D | D | C | C | C |
| SUPERVISOR | D | D | D | C | C | C | B |
| OFFICER/EXECUTIVE | D | D | C | C | C | B | B |
| SR.OFFICER | D | C | C | C | B | B | B |
| PROFESSIONAL | C | C | C | B | B | B | A |

Table S1. The matrix developed to categorise the respondents into High, middle and low socio-economic groups ([www.praja.org](http://www.praja.org))

A/B – High socio-economic group; C/D – Middle socio-economic group; E/F – Low socio-economic group

| **Questionnaire** | **n (%)**** | **Age of respondents** | | **P**  **value** | **Family size** | | **P value** | **Children<14years** | | **P value** | **Social status** | | **P value** |
| --- | --- | --- | --- | --- | --- | --- | --- | --- | --- | --- | --- | --- | --- |
|  |  | **≤34years** | **≥35 years** |  | **≤5** | **≥6** |  | **Yes** | **No** |  | **Others @** | **Lower** |  |
| Can cats transmit rabies? | |  |  | 0.09 |  |  | 0.42 |  |  | 0.37 |  |  | 0.8 |
| Yes | 27 (22) | 17 | 10 |  | 15 | 12 |  | 14 | 13 |  | 15 | 12 |  |
| No | 96 (78) | 43 | 53 |  | 45 | 51 |  | 59 | 37 |  | 56 | 40 |  |
| Can rats transmit rabies? | | |  | 0.5 |  |  | 0.28 |  |  | 0.65 |  |  | 0.88 |
| Yes | 22 (18) | 12 | 10 |  | 13 | 9 |  | 14 | 8 |  | 13 | 9 |  |
| No | 101 (82) | 53 | 48 |  | 47 | 54 |  | 59 | 42 |  | 58 | 43 |  |
| Can rabies be transmitted through animal bites? | | | | 0.5# |  | | 0.49# |  | | 0.79# |  | | 0.18# |
| Yes | 121 (98) | 60 | 61 |  | 61 | 60 |  | 72 | 49 |  | 71 | 50 |  |
| No | 2 (2) | 0 | 2 |  | 2 | 0 |  | 1 | 1 |  | 0 | 2 |  |
| Can rabies be transmitted through licks/scratches? | | | | 0.5 |  | | 0.88 |  | | 0.29 |  | | 0.99 |
| Yes | 50 (41) | 26 | 24 |  | 24 | 26 |  | 28 | 22 |  | 34 | 16 |  |
| No | 73 (59) | 34 | 39 |  | 36 | 37 |  | 45 | 28 |  | 37 | 36 |  |
| Is rabies fatal |  |  |  | 0.5# |  |  | 0.08# |  |  | 0.26 |  |  | 0.67 |
| Yes | 106 (86) | 53 | 53 |  | 55 | 51 |  | 65 | 41 |  | 62 | 44 |  |
| No | 17 (14) | 7 | 10 |  | 5 | 12 |  | 8 | 9 |  | 9 | 8 |  |
| Can rabies be prevented? | | | | 0.4 |  |  | 0.9 |  |  | 0.32 |  |  | 0.42 |
| Yes | 98 (80) | 46 | 52 |  | 48 | 50 |  | 56 | 42 |  | 54 | 44 |  |
| No | 25 (20) | 14 | 11 |  | 12 | 13 |  | 17 | 8 |  | 16 | 9 |  |
| Can rabies be prevented by post-bite anti-rabies vaccines (ARV)? | | | | 0.96 |  | | 0.71 |  | | 0.7 |  | | 0.4 |
| Yes | 90 (73) | 44 | 46 |  | 47 | 43 |  | 52 | 38 |  | 54 | 36 |  |
| No | 33 (27) | 16 | 17 |  | 16 | 17 |  | 21 | 12 |  | 17 | 16 |  |
| Can rabies be prevented by vaccinating dogs against rabies? | | | | 0.96 |  | | **0.01** |  | | 0.22 |  | | 0.22 |
| Yes | 90 (73) | 44 | 46 |  | 50 | 40 |  | 50 | 40 |  | 49 | 41 |  |
| No | 33 (27) | 16 | 17 |  | 10 | 23 |  | 23 | 10 |  | 22 | 11 |  |

Table S2. Bivariate analyses of responses to the questions pertaining to knowledge of rabies for various descriptive determinants. *

Continued/

| **Questionnaire** | **n (%)**** | **Dog ownership** | | **P value** | **Cattle/buffalo ownership** | | **P value** | **Sheep/goat ownership** | | **P value** | **Poultry ownership** | | **P value** |
| --- | --- | --- | --- | --- | --- | --- | --- | --- | --- | --- | --- | --- | --- |
|  |  | **Yes** | **No** |  | **Yes** | **No** |  | **Yes** | **No** |  | **Yes** | **No** |  |
| Can cat transmit rabies? |  |  |  | 0.9 |  |  | 0.57 |  |  | 0.81 |  |  | 0.6 |
| Yes | 27 (22) | 14 | 13 |  | 16 | 11 |  | 17 | 10 |  | 17 | 10 |  |
| No | 96 (78) | 51 | 45 |  | 51 | 45 |  | 58 | 38 |  | 15 | 41 |  |
| Can rat transmit rabies? |  |  |  | 0.99 |  |  | 0.63 |  |  | 0.81 |  |  | 0.15# |
| Yes | 22 (18) | 12 | 10 |  | 13 | 9 |  | 14 | 8 |  | 16 | 6 |  |
| No | 101 (82) | 53 | 48 |  | 54 | 47 |  | 61 | 40 |  | 56 | 45 |  |
| Can be transmitted through animal bites? | | | | 0.22# | 0.99# | | | 0.52# | | | 0.16# | | |
| Yes | 121 (98) | 65 | 56 |  | 66 | 55 |  | 73 | 48 |  | 72 | 49 |  |
| No | 2 (2) | 0 | 2 |  | 1 | 1 |  | 2 | 0 |  | 0 | 2 |  |
| Can be transmitted through licks/scratches? | | | | 0.09 | **0.004** | | | **0.004** | | | **0.03** | | |
| Yes | 50 (41) | 31 | 19 |  | 35 | 15 |  | 38 | 12 |  | 35 | 15 |  |
| No | 73 (59) | 34 | 39 |  | 32 | 41 |  | 12 | 36 |  | 37 | 36 |  |
| Is rabies fatal? |  |  |  | 0.6 |  |  | 0.99 |  |  | 0.99# |  |  | 0.6 |
| Yes | 106 (86) | 57 | 49 |  | 58 | 48 |  | 65 | 41 |  | 63 | 43 |  |
| No | 17 (14) | 8 | 9 |  | 9 | 8 |  | 10 | 7 |  | 9 | 8 |  |
| Can rabies be prevented? |  |  |  | 0.31 |  |  | 0.72 |  |  | 0.78 |  |  | 0.77 |
| Yes | 98 (80) | 54 | 44 |  | 59 | 39 |  | 54 | 44 |  | 58 | 40 |  |
| No | 25 (20) | 11 | 14 |  | 16 | 9 |  | 13 | 12 |  | 14 | 11 |  |
| Can rabies be prevented by post -bite anti-rabies vaccines (ARV)? | | | | 0.5 | 0.22 | | | 0.03 | | | 0.17 | | |
| Yes | 90 (73) | 49 | 41 |  | 52 | 38 |  | 60 | 30 |  | 56 | 34 |  |
| No | 33(27) | 16 | 17 |  | 15 | 18 |  | 15 | 18 |  | 16 | 17 |  |
| Can rabies be prevented by vaccinating dogs against rabies? | | | | 0.07 | 0.99 | | | 0.22 | | | 0.26 | | |
| Yes | 90 (73) | 52 | 38 |  | 46 | 44 |  | 52 | 38 |  | 50 | 40 |  |
| No | 33 (27) | 13 | 20 |  | 21 | 12 |  | 23 | 10 |  | 22 | 11 |  |

*Only those respondents who answered yes (n=123) to “have you heard of rabies?” were administered rest of the questionnaire. All (n=123) respondents answered “yes” to “Is rabies transmitted through dogs?” ; and answered “Dogs” , when asked “ Which is the most common animal responsible for bite wounds?” ** The bivariate analyses were based on the responses from 123 participants who had heard of rabies; # Fisher’s exact test. @ Others include High/Middle socio-economic group. Significant p values are denoted in bold.

Table S3. Bivariate analyses of responses to the questions pertaining to attitudes and practices regarding rabies for various descriptive determinants.

| **Questionnaire** | | **n (%)*** | **Age of**  **respondents** | | **P value** | **Family size** | | **P value** | **Children**  **<14years** | | **P value** | **Social status** | | **P value** |
| --- | --- | --- | --- | --- | --- | --- | --- | --- | --- | --- | --- | --- | --- | --- |
|  |  |  | **≤34years** | **≥35years** |  | **≤5** | **≥6** |  | **Yes** | **No** |  | **Others@** | **Lower** |  |
| In your opinion will the application of local treatments, like chilli powder and turmeric, on animal bites wounds prevent rabies? | | |  | | | | | | | | | | | |
| Yes |  | 20 (16) | 12 | 8 | 0.3 | 9 | 11 | 0.65 | 11 | 9 | 0.7 | 8 | 12 | 0.99 |
| No |  | 107 (84) | 51 | 56 |  | 54 | 53 |  | 63 | 44 |  | 44 | 63 |  |
| In your opinion should animal bite wounds be washed with soap and water to reduce the chances of rabies infection? | | |  | | | | | | | | | | | |
| Yes |  | 52 (41) | 21 | 31 | 0.08 | 24 | 28 | 0.51 | 33 | 19 | 0.32 | 23 | 29 | 0.53 |
| No |  | 75 (59) | 42 | 33 |  | 39 | 36 |  | 41 | 34 |  | 29 | 46 |  |
| In your opinion is it necessary to go to a hospital if someone is bitten by a dog, even if the injury is not severe? | | |  | | | | | | | | | | | |
| Yes |  | 119 (94) | 57 | 62 | 0.16# | 58 | 61 | 0.5# | 70 | 49 | 0.71# | 50 | 69 | 0.46# |
| No |  | 8 (6) | 6 | 2 |  | 5 | 3 |  | 4 | 4 |  | 2 | 6 |  |
| Can rabies be controlled by restricting the size of the stray dog population? | | |  | | | | | | | | | | | |
| Yes |  | 113 (89) | 57 | 56 | 0.77# | 57 | 56 | 0.59# | 68 | 45 | 0.21# | 46 | 67 | 0.99# |
| No |  | 14 (11) | 6 | 8 |  | 6 | 8 |  | 6 | 8 |  | 6 | 8 |  |
| If you saw a dog with signs of rabies would you inform the municipal authorities? | |  |  | | | | | | | | | | | |
| Yes |  | 90 (71) | 41 | 49 | 0.15 | 40 | 50 | 0.19 | 54 | 36 | 0.53 | 40 | 50 | 0.21 |
| No |  | 37 (29) | 22 | 15 |  | 12 | 25 |  | 20 | 17 |  | 12 | 25 |  |

Continued/

| **Questionnaire** | | **n (%)*** | **Dog ownership** | | **P value** | **Cattle/buffalo ownership** | | **P value** | **Sheep/goat ownership** | | **P**  **value** | **Poultry ownership** | | **P value** |
| --- | --- | --- | --- | --- | --- | --- | --- | --- | --- | --- | --- | --- | --- | --- |
|  |  |  | **Yes** | **No** |  | **Yes** | **No** |  | **Yes** | **No** |  | **Yes** | **No** |  |
| In your opinion will the application of local treatments, like chilli powder and turmeric, on animal bites wounds prevent rabies? | | |  | | | | | | | | | | | |
| Yes |  | 20 (16) | 12 | 8 | 0.62 | 10 | 10 | 0.72 | 13 | 7 | 0.8# | 9 | 11 | 0.18 |
| No |  | 107 (84) | 55 | 52 |  | 58 | 49 |  | 64 | 43 |  | 65 | 42 |  |
| In your opinion should animal bite wounds be washed with soap and water to reduce chances of rabies infection? | | |  | | | | | | | | | | | |
| Yes |  | 52 (41) | 23 | 29 | 0.1 | 32 | 20 | 0.13 | 38 | 14 | **0.01** | 32 | 42 | 0.53 |
| No |  | 75 (59) | 44 | 31 |  | 36 | 39 |  | 39 | 36 |  | 20 | 33 |  |
| In your opinion is it necessary to go to hospital if someone is bitten by a dog, even if the injury is not severe? | | |  | | | | | | | | | | | |
| Yes |  | 119 (94) | 63 | 56 | 0.99# | 65 | 54 | 0.34# | 74 | 45 | 0.16# | 69 | 50 | 0.99# |
| No |  | 8 (6) | 4 | 4 |  | 3 | 5 |  | 3 | 5 |  | 5 | 3 |  |
| Can rabies be controlled by restricting the size of the stray dog population? | | |  | | | | | | | | | | | |
| Yes |  | 113 (89) | 59 | 54 | 0.8# | 62 | 51 | 0.41# | 71 | 42 | 0.16# | 69 | 44 | 0.08# |
| No |  | 14 (11) | 8 | 6 |  | 6 | 8 |  | 6 | 8 |  | 5 | 9 |  |
| If you saw a dog with signs of rabies would you inform the municipal authorities? | |  | | | | | | | | | | | | |
| Yes |  | 90 (71) | 47 | 43 | 0.85 | 46 | 44 | 0.4 | 51 | 39 | 0.15 | 46 | 44 | **0.01** |
| No |  | 37 (29) | 20 | 17 |  | 22 | 15 |  | 26 | 11 |  | 28 | 9 |  |

* The bivariate analysis was based on responses from all (n=127) respondents. @ Others include High/Middle socio-economic group. Significant p values are denoted in bold.

Table S4. Bivariate analyses of the response by various categories of respondents to the questions regarding their attitudes and practices against free roaming dogs.

| **Questionnaire** | | **n (%)*** | **Age**  **of respondents** | | **P value** | **Family**  **size** | | **P value** | **Children**  **<14years** | | **P**  **value** | **Social status** | | **P value** |
| --- | --- | --- | --- | --- | --- | --- | --- | --- | --- | --- | --- | --- | --- | --- |
|  |  |  | **≤34years** | **≥35years** |  | **≤5** | **≥6** |  | **Yes** | **No** |  | **Others@** | **Lower** |  |
| Do you feel FRD in your locality are useful to society? | | |  | | | | | | | | | | | |
| Yes |  | 17 (13) | 9 | 8 | 0.8 | 6 | 11 | 0.2# | 7 | 10 | 0.99# | 11 | 6 | **0.03#** |
| No |  | 110 (87) | 55 | 55 |  | 58 | 52 |  | 65 | 45 |  | 41 | 69 |  |
| Do you think that the FRD in your locality are a nuisance or a problem for the society? | | |  | | | | | | | | | | | |
| Yes |  | 109 (86) | 53 | 56 | 0.44# | 57 | 52 | 0.32# | 46 | 63 | 0.99# | 68 | 41 | 0.07# |
| No |  | 18 (14) | 11 | 7 |  | 7 | 11 |  | 7 | 11 |  | 7 | 11 |  |
| Do you think that the FRD are a threat to human health? | | |  | | | | | | | | | | | |
| Yes |  | 116 (91) | 55 | 61 | **0.05**# | 57 | 52 | 0.75# | 69 | 47 | 0.5# | 68 | 48 | 0.33# |
| No |  | 11 (9) | 9 | 2 |  | 7 | 11 |  | 5 | 6 |  | 7 | 4 |  |
| Do you ever feed FRD? | | |  | | | | | | | | | | | |
| Yes |  | 50 (39) | 31 | 19 | **0.04** | 28 | 22 | 0.2 | 26 | 24 | 0.24 | 28 | 22 | **0.005** |
| No |  | 77 (61) | 33 | 44 |  | 35 | 42 |  | 48 | 29 |  | 24 | 53 |  |
| If you see an injured FRD would you take it to a veterinarian? | | |  | | | | | | | | | | | |
| Yes |  | 28 (22) | 17 | 11 |  | 18 | 10 |  | 14 | 14 |  | 16 | 12 |  |
| No |  | 99 (78) | 47 | 52 | 0.21 | 45 | 54 | 0.07 | 60 | 39 | 0.31 | 36 | 63 | **0.04** |
| In your opinion should people who feed / shelter these dogs take responsibility for their health and vaccination? | | |  | | | | | | | | | | | |
| Yes |  | 34 (27) | 16 | 18 |  | 18 | 16 |  | 21 | 13 |  | 15 | 19 |  |
| No |  | 93 (73) | 48 | 45 | 0.64 | 45 | 48 | 0.64 | 53 | 40 | 0.62 | 60 | 33 | **0.03** |
| In your opinion is it the responsibility of the government to take care of the health of FRD? | | |  |  |  |  |  |  |  |  |  |  |  |  |
|  |  |  |  |  |  |  |  |  |  |  |  |  |  |  |
| Yes |  | 119 (94) | 61 | 58 | 0.49# | 59 | 60 | 0.99# | 70 | 49 | 0.71# | 50 | 69 | 0.34# |
| No |  | 8 (6) | 3 | 5 |  | 4 | 4 |  | 4 | 4 |  | 2 | 6 |  |

Continued/-

| **Questionnaire** | | **n* (%)** | **Dog ownership** | | **P value** | **Cattle/buffalo ownership** | | **P value** | **Sheep/goat ownership** | | **P value** | **Poultry ownership** | | **P value** |
| --- | --- | --- | --- | --- | --- | --- | --- | --- | --- | --- | --- | --- | --- | --- |
|  |  |  | **Yes** | **No** |  | **Yes** | **No** |  | **Yes** | **No** |  | **Yes** | **No** |  |
| Do you feel FRD in your locality are useful to society? | |  |  |  |  |  |  |  |  |  |  |  |  |  |
| Yes |  | 17 (13) | 6 | 11 | 0.31# | 6 | 11 | 0.12# | 10 | 7 | 0.1# | 8 | 9 | 0.79 |
| No |  | 110 (87) | 54 | 56 |  | 62 | 48 |  | 40 | 70 |  | 45 | 65 |  |
| Do you think that the FRD in your locality are a nuisance or a problem for the society? | | |  | | | | | | | | | | | |
| Yes |  | 109 (86) | 55 | 54 | 0.3# | 61 | 48 | 0.2# | 71 | 38 | **0.01**# | 66 | 43 | 0.2 |
| No |  | 18 (14) | 12 | 6 |  | 7 | 11 |  | 6 | 12 |  | 8 | 10 |  |
| Do you think that the FRD are a threat to human health? | |  |  | | | | | | | | | | | |
| Yes |  | 116 (91) | 59 | 57 | 0.21# | 64 | 52 | 0.34# | 72 | 44 | 0.33 | 69 | 47 | 0.52# |
| No |  | 11 (9) | 8 | 3 |  | 4 | 7 |  | 5 | 6 |  | 5 | 6 |  |
| Do you ever feed FRD? | |  |  |  |  |  |  |  |  |  |  |  |  |  |
| Yes |  | 50 (39) | 24 | 26 | 0.38 | 17 | 33 | **0.003** | 27 | 23 | **0.003** | 27 | 23 | 0.4 |
| No |  | 77 (61) | 43 | 34 |  | 51 | 26 |  | 50 | 27 |  | 47 | 30 |  |
| If you see an injured FRD would you take it to a veterinarian? | |  |  |  |  |  |  |  |  |  |  |  |  |  |
| Yes |  | 28 (22) | 17 | 11 | 0.33 | 16 | 12 | 0.66 | 16 | 12 | 0.66 | 16 | 12 | 0.89 |
| No |  | 99 (78) | 50 | 49 |  | 52 | 47 |  | 61 | 38 |  | 58 | 41 |  |
| In your opinion should people who feed / shelter these dogs take responsibility for their health and vaccination? | | |  | | | | | | | | | | | |
| Yes |  | 34 (27) | 13 | 21 | **0.04** | 14 | 20 | 0.09 | 17 | 17 | 0.13 | 18 | 16 | 0.46 |
| No |  | 93 (73) | 54 | 39 |  | 54 | 39 |  | 60 | 33 |  | 56 | 37 |  |
| In your opinion is it the responsibility of the government to take care of the health of FRD? | | |  | | | | | | | | | | | |
| Yes |  | 119 (94) | 62 | 57 | 0.72# | 64 | 55 | 0.9# | 74 | 45 | 0.16# | 70 | 49 | 0.71# |
| No |  | 8 (6) | 5 | 3 |  | 4 | 4 |  | 3 | 5 |  | 4 | 4 |  |

* The bivariate analyses was based on responses from all (n=127) respondents. # Fisher exact test. @ Others include High/Middle socio-economic group. Significant p values are denoted in bold.
