# Supplementary material for: Knowledge, attitudes and practices (KAP) towards rabies and free-roaming dogs (FRD) in Shirsuphal village in western India: A community based cross-sectional study
Source: PLoS Negl Trop Dis. 2019 Jan 25;13(1):e0007120. doi: 10.1371/journal.pntd.0007120 (PMC6364945; doi:10.1371/journal.pntd.0007120)
Supplement: S2 File — (PDF) [file pntd.0007120.s002.pdf]

Murdoch UNIVERSITY

Division of Research & Development
Research Ethics and Integrity

Chancellery Building
South Street
MURDOCH WA 6150
Telephone: (08) 9360 6677
Facsimile: (08) 9360 6686
human.ethics@murdoch.edu.au

www.murdoch.edu.au

Thursday, 05 May 2016

Prof Ian Robertson
School of Veterinary and Life Sciences
Murdoch University

Dear Ian,

**Project No.**	2016/020
**Project Title**	Studies on the population dynamics of free roaming stray dogs and owned dogs in Mumbai and Guwahati

Thank you for addressing the conditions placed on the above application to the Murdoch University Human Research Ethics Committee. On behalf of the Committee, I am pleased to advise the application now has:

OUTRIGHT APPROVAL

Approval is granted on the understanding that research will be conducted according the standards of the ***National Statement on Ethical Conduct in Human Research*** **(2007), the *Australian Code for the Responsible Conduct of Research*** **(2007)** and **Murdoch University policies** at all times. You must also abide by the **Human Research Ethics Committee's standard conditions of approval (see attached).** All reporting forms are available on the Research Ethics and Integrity web-site.

I wish you every success for your research.

Please quote your ethics project number in all correspondence.

Kind Regards,

E. von Dietze

Dr. Erich von Dietze
Manager
Research Ethics and Integrity

cc: Dr Mark O'Dea and Harish Tiwari

Human Research Ethics Committee: Standard Conditions of Approval

a) The project must be conducted in accordance with the approved application, including any conditions and amendments that have been approved. You must comply with all of the conditions imposed by the HREC, and any subsequent conditions that the HREC may require.

b) You must report immediately anything, which might affect ethical acceptance of your project, including:
   - *Adverse effects on participants*
   - *Significant unforeseen events*
   - *Other matters that might affect continued ethical acceptability of the project.*

c) Proposed changes or amendments to the research must be applied for, using an Amendment Application form, and approved by the HREC before these may be implemented.

d) An Annual Report for the project must be provided by the due date specified each year (usually the anniversary of approval).

e) A Closure Report must be provided at the conclusion of the project (once all contact with participants has been completed).

f) If, for any reason, the project does not proceed or is discontinued, you must advise the committee in writing, using a Closure Report form.

g) If an extension is required beyond the end date of the approved project, an Extension Application should be made allowing sufficient time for its consideration by the committee. Extensions of approval cannot be granted retrospectively.

h) You must advise the HREC immediately, in writing, if any complaint is made about the conduct of the project.

i) Other Murdoch approvals (e.g. fieldwork approval) or approval form other institutions may also be necessary before the research can commence.

j) Any equipment used must meet current safety standards. Purpose built or modified equipment must be tested and certified by independent experts for compliance with safety standards.

k) Graduate research degree candidates must normally have their Program of Study approved prior to commencing the research. Exceptions to this must be approved by the HREC.

l) You must notify Research Ethics & Integrity of any changes in contact details including address, phone number and email address.

m) Researchers should be aware that the HREC may conduct random audits and / or require additional reports concerning the research project.

Failure to comply with the *National Statement on Ethical Conduct in Human Research* (2007) and with the conditions of approval may result in the suspension or withdrawal of approval for the project.

The HREC seeks to support researchers in achieving strong results and positive outcomes.

The HREC promotes a research culture in which ethics is considered and discussed at all stages of the research.

If you have any issues you wish to raise, please contact the Research Ethics Office in the first instance.

Survey questionnaire for urban and rural respondents including pet dog owners to determine knowledge, attitudes and practices of the community towards dogs and the control of rabies

1. **Household Information**

1.1	Name of the respondent	
1.2	Gender	Male_____Female _____
1.3	Age	
1.4	How many people including yourself live in this household?	
1.5	How many children aged 14 years or less live in this household?	
1.6	What is your highest educational qualification?<br>Please mark ‘×’ against the level of education.	No formal education ____<br>Primary school _________<br>Secondary school ________<br>Matriculation___________<br>College____________<br>Graduate_____________<br>Post- Graduation<br>_________
1.7	How would you describe your occupation?<br>Please mark ‘x’ against suitable option.	Unskilled work _________<br>Skilled work__________<br>Small trading ____________<br>Shop owner__________<br>Business as self-employed professional___________<br>Businessmen___________<br>Clerical/salesman_______<br>Supervisory level________<br>Officer/executives______<br>Senior officers__________<br>Others, Please specify _____
1.6	What religion are you?	Hindu ( )<br>Islam ( )<br>Buddhist ( )<br>Christian( )<br>Prefer not to say ( )<br>No religion ( )
1.9	Do you own any pet animal(s)? If no, proceed to 1.11	Yes ( ) No ( )
1.10	What pets do you own?	Dog( )<br>Cat ( )<br>Other, please specify<br>( )
1.11	Do you own any livestock? If no, proceed to section 2	Yes ( )<br>No ( )
1.12	What kind and number of livestock do you own?	Cattle/buffaloes ( )<br>Horses ( )<br>Pigs ( )<br>Sheep/goats ( )<br>Poultry ( )

2. Details of ward and garbage disposal

2.1	Would you rank the garbage disposal system of your ward as satisfactory or unsatisfactory?	Satisfactory ( )<br>unsatisfactory ( )
2.2	Would you rank the general cleanliness of open areas and streets of your ward as satisfactory or unsatisfactory?	Satisfactory ( )<br>unsatisfactory ( )

3. Knowledge of rabies

3.1	Have you ever heard of the disease called rabies?<br>If no proceed to section 4.	Yes ( ) No ( )
3.2	In your opinion can dogs transmit rabies?	Yes ( ) No ( )<br>Not sure ( )
3.3	In your opinion can cats transmit rabies?	Yes ( ) No ( )<br>Not sure ( )
3.4	In your opinion can rats transmit rabies?	Yes ( ) No ( )<br>Not sure ( )
3.5	In your opinion which animal most commonly causes rabies?	Don't know ( ) Dogs ( )<br>Cats ( ) Rats ( )<br>Monkeys ( ) wild life ( )<br>Others, please specify<br>( )
3.6	In your opinion can animal bites transmit rabies?	Yes ( ) No ( )<br>Not sure ( )
3.7	In your opinion can licks/scratches from animals transmit rabies?	Yes ( ) No ( )<br>Not sure ( )
3.8	Do you think rabies is fatal if symptoms appear?	Yes ( ) No ( )<br>Not sure ( )
3.9	In your opinion can rabies be prevented?	Yes ( ) No ( )<br>Not sure ( )
3.10	In your opinion will application of local treatments, like chilli powder and turmeric, on animal bite wounds prevent rabies?	Yes ( ) No ( )<br>Not sure ( )
3.11	In your opinion should an animal bite wound be washed with soap and water to reduce chances of rabies infection?	Yes ( ) No ( )<br>Not sure ( )
3.12	In your opinion is it necessary to go to hospital if someone is bitten by a dog, even if the injury is not severe?	Yes ( ) No ( )<br>Not sure ( )
3.13	Are you aware that post-bite anti-rabies vaccines can prevent rabies in humans?	Yes ( ) No ( )<br>Not sure ( )
3.14	Can rabies be prevented by vaccinating dogs against the disease?	Yes ( ) No ( )<br>Not sure ( )
3.15	Can rabies be controlled by restricting the size of the stray dog population?	Yes ( ) No ( )<br>Not sure ( )
3.16	If you saw a dog with signs of rabies would you inform the municipal authorities?	Yes ( ) No ( )<br>Not sure ( )
3.17	Have any awareness campaigns been organised in your locality during the last two years about rabies and how to control it?	Yes ( ) No ( )<br>Not sure ( )

4. Free roaming dogs

4.1	Are there any free roaming dogs in your locality?<br>If yes proceed to next question.<br>If no and you have pet dogs, please proceed to section 5.<br>If no and you do not own pet dogs, the questionnaire for you ends here. Thanks for your time to complete this questionnaire.	Yes ( ) No ( )<br>Not sure ()
4.2	Where do you think these free roaming dogs come from?	From nearby localities ( )<br>Breeding of local dogs ( )<br>Abandoned pet dogs ( )<br>Others, please specify<br>( )<br>Not Sure ( )
4.3	Approximately how many free roaming dogs do you think are present in your locality?	
4.4	Do you feel the free roaming dogs in your locality are useful to society? If no, proceed to 4.6	Yes ( ) No ( )<br>Not sure ( )
4.5	What do you think are the benefits of free roaming dogs?	Guarding ( )<br>Keep away wild animals( )<br>Keep away thieves ( )<br>Other, please specify<br>( )
4.6	Do you believe the free roaming dogs in your locality are a nuisance or a problem for the society?	Yes ( ) No ( )<br>Not sure ( )
4.7	Do you think that the free roaming dogs in your locality are a threat to human health?	Yes ( ) No ( )<br>Not sure ( )
4.8	Where do you think these dogs get their food?	Fed by residents ( )<br>Garbage dumps ( )<br>Litter from streets ( )<br>Other, please specify ( )<br>Not sure ( )
4.9	Do you ever feed free roaming stray dogs? If yes, proceed to next question, else go to 4.13	Yes ( ) No ( )<br>Not Sure( )
4.10	Do you think feeding of free roaming dogs is part of your religious duties?	Yes ( ) No ( )<br>Not sure ( )
4.11	Do you think feeding of free roaming dogs is an act of love/ compassion towards these animals?	Yes ( ) No ( )<br>Not sure ( )
4.12	Do you think feeding of free roaming dogs is better than wasting the food?	Yes ( ) No ( )<br>Not sure ( )
4.13	Would you rank the health of the free roaming dogs in your locality as good, average or poor?	Good health ( )<br>Average health( )<br>Poor health ( )
4.14	If you see an injured free roaming stray dog, would you take it to a veterinarian?	Yes ( ) No ( )<br>Not sure ( )
4.15	In your opinion should people who feed / shelter these dogs take responsibility for their health and vaccination?	Yes ( ) No ( )<br>Not sure ( )
4.16	In your opinion is it the responsibility of the Government to take care of the health of free roaming dogs?	Yes ( ) No ( )<br>Not sure ( )

4.17	Are you aware of any programmes undertaken in the last two years in your area to control the dog population?	Yes ( ) No ( )<br>Not sure ( )
4.18	In your opinion which of the following is the best way to control the free roaming free dog population? Culling; Impounding; birth control operations; better garbage management; if any other, please specify	Culling ( )<br>Impounding ( )<br>birth control operations( )<br>better garbage management ( )<br>if any other, please specify ( )<br>Not sure ( )

5. **Dog owning population (only for pet dog owners)**

5.1	How many dogs do you own?	Male________<br>Female ___________<br>Total __________
5.2	What is their age /ages?	
5.3	What is the breed of your pet dogs?	Local ( )<br>Pedigreed( ), specify<br>____________<br>Mixed ( )
5.4	Where did you get your pet from?	Purchased ( )<br>Gifted ( )<br>Adopted ( )<br>Offspring of owned bitch ( )<br>Other, please specify ( )
5.5	Would you prefer to own a pedigreed pup to a local Indian Native dog? If yes, proceed to next question, else to 5.7	Yes ( )<br>No ( )<br>Not sure ( )
5.6	What are the reasons for you to prefer pedigreed dogs rather than local Indian Native dogs?	Social status ( )<br>Intelligence of dogs( )<br>Cleanliness ( )<br>Other reasons, please specify ( )<br>No specific reason ( )
5.7	Is your dog always confined to your home premises? If yes, proceed to next question, else to 5.9	Yes ( ) No ( )
5.8	When away from your house premises, does your dog accompany you or members of your household (a) always (b) sometimes or (c) rarely (d) never	
5.9	Is your pet registered?	Yes ( ) No ( )<br>Applied for ( )<br>Not sure ( )
5.10	In the last year have you taken your dog to a veterinarian? If no proceed to 5.13	Yes ( )<br>No ( )
5.11	How many times was your dog taken to the veterinarian in the last year?	
5.12	Has your dog ever been vaccinated against rabies? If not vaccinated, proceed to 5.14	Yes ( ) No ( )<br>Not sure ( )

5.13	If vaccinated, when was the last vaccine given?	
5.14	Has your dog been operated upon so that it cannot breed?	Yes ( )<br>No ( )<br>Don't Know ( )
5.15	If your dog has not been operated to stop breeding, is there a reason for this?	Sterilising cost ( )<br>Lack of service ( )<br>Not aware of such procedures ( )<br>Breeding purpose ( )<br>Cruel practice ( )<br>Religion ( )<br>Other, please specify( )<br>No reason ( )

Thank you for your time to complete this questionnaire. This information will help understand the role of dogs in the community and the control of rabies.

पाळीव कुत्रांचे मालक तसेच शहरी व ग्रामीण लोकांची रेबीज थांबवण्याबददलची माहिती, दृष्टीकोन आणि पद्धती या साठीची प्रश्नावली

१. घरघुती माहिती

१.१	लिंग	पुरूष / स्त्री
१.२	वय	
१.३	घरात तुम्हाला स्वतःला धरून किती माणसे आहेत?	
१.४	कुटूंबात १४ वर्षाखालील किती मुले आहेत?	
१.५	शैक्षणिक पात्रता	अशिक्षित<br>प्राथमिक शिक्षण<br>माध्यमिक शिक्षण<br>उच्च माध्यमिक शिक्षण<br>पदवी<br>पदविका<br>परव्युत्तर
१.६	काम /व्यवसायाची माहिती	अकुशल<br>कुशल<br>लघु व्यवसाय<br>दुकानदार<br>स्वतःचा व्यवसाय<br>व्यवसायीक<br>व्यापारी<br>लिपीक कामगार<br>मिस्त्री / ठेकेदार<br>अधिकारी<br>उच्च अधिकारी<br>इरत

१.७	धर्म	हिंदु<br>मुस्लिम<br>ख्रिश्चन<br>बौद्ध<br>सांगु इच्छीत नाही<br>धर्म नाही
१.८	तुमच्याकडे पाळीव प्राणी आहे का?<br>जर नाही तर मुद्दा क्र १.११ वर जा.	होय / नाही
१.९	तुमच्याकडे कोणते पाळीव प्राणी आहेत?	कुत्रा<br>मांजर<br>इतर
१.१0	तुमच्याकडे कोण कोणती जनावरे आहेत?<br>जर नाही तर मुद्दा क्र २ वर जा.	होय / नाही
१.११	तुमच्याकडे कोणती जनावरे आहेत ?<br>आणि किती आहेत ?	गाई<br>म्हैस<br>घोडा<br>डुक्कर<br>शेळी/ मेंढी<br>कोंबडया

२. कचऱ्याच्या व्यवस्थापनाबददल माहिती

२.१	तुमच्या गावातल्या कचरा व्यवस्थापनाबददल तुम्ही समाधानी आहात का?	होय / नाही
२.२	तुमच्याकडील रस्ते व मोकळया जागेच्या स्वच्छतेबददल तुम्ही समाधानी आहात का?	होय / नाही

३. रेबीज बदद्लची माहिती

३.१	तुम्हाला रेबीज बदद्ल माहिती आहे का? जर नाही तर मुद्दा क्र १.११ वर जा.	होय / नाही / माहित नाही
३.२	कुत्र्यामुळे रेबीज होतो का?	होय / नाही / माहित नाही
३.३	मांजरामुळे रेबीज होतो का?	होय / नाही / माहित नाही
३.४	उंदरामुळे रेबीज होतो का?	होय / नाही / माहित नाही
३.५	कोणत्या प्राण्यांमध्ये रेबीज जास्त दिसुन येतो?	
३.६	प्राणी चावल्यामुळे रेबीजचा प्रसार होउ शकतो का?	होय / नाही / माहित नाही
३.७	प्राण्यांच्या चाटण्यामुळे किंवा बोचकरल्याने रेबीजचा प्रसार होउ शकतो का?	होय / नाही / माहित नाही
३.८	रेबीजची लक्षणे दिसल्यावर मृत्य होतो असे तुम्हाला वाटते का?	होय / नाही / माहित नाही
३.९	रेबीजवरती नियंत्रण ठेवता येऊ शकतो का?	होय / नाही / माहित नाही
३.१0	स्थानिक उपचार जसे की, मिर्ची पुड किंवा हळद जखमेवर लावल्यास रेबीज होत नाही असे तुम्हाला वाटते का?	होय / नाही / माहित नाही
३.११	प्राणी चावल्याच्या जखमेवर साबन लावुन पाण्याने धुतल्यास रेबीज होण्याची शक्यता कमी करता येईल असे तुम्हाला वाटते का?	होय / नाही / माहित नाही
३.१२	जखम जरी खोल नसली तरी कुत्रा चावल्यावर दवाखान्यात जाणे गरजेचे आहे का?	होय / नाही / माहित नाही
३.१३	कुत्रा चावल्यावर रेबीज प्रतीबंधक लस माणसा मधला रेबीज प्रतीबंधीत करू शकते का?	होय / नाही / माहित नाही
३.१४	कुत्र्यांना रेबीज प्रतीबंधक लस दिल्यामुळे रेबीज प्रतीबंध होऊ शकतो का?	होय / नाही / माहित नाही
३.१५	मोकाट कुत्र्यांची संख्या प्रतीबंधीत केल्यामुळे रेबीजचा प्रतीबंध होऊ शकतो का?	होय / नाही / माहित नाही
३.१६	तुम्हाला रेबीज (पिसाळलेला) कुत्रा दिसुन आल्यास संबंधीत अधिकाऱ्याला माहिती दयाल का?	होय / नाही / माहित नाही
३.१७	तुमच्या गावामध्ये गेल्या दोन वर्षात रेबीजवर नियंत्रण ठेवण्यासाठी कुठली मोहीम किंवा कार्यक्रम झाला आहे का?	होय / नाही / माहित नाही

४.भटक्या कुत्र्यांविषयी माहिती

४.१	तुमच्या गावामध्ये भटके कुत्रे आहेत का? असल्यास पुढील प्रश्न सोडवा.<br>जर नाही आणि तुमच्याकडे पाळीव कुत्रे आहे तर मुद्दा क्र ५ वर जा.<br>जर दोन्हींपैकी काहीही नाही तर आपल्यासाठी ही प्रश्नावली येथेच संपते.<br>तुमचा अमुल्य वेळ दिल्याबददल धन्यवाद.	होय / नाही / माहित नाही
४.२	भटके कुत्रे कोठुन येता असे तुम्हाला वाटते?	शेजारच्या गावातुन<br>इथल्या कुत्र्यांच्या प्रजननातुन<br>नियंत्रन नसलेल्या पाळीव कुत्र्यांमुळे<br>इतर<br>माहीत नाही
४.३	तुमच्या गावातील भटक्या कुत्र्यांची संख्या अंदाजे किती असेल?	
४.४	गावातील भटकी कुत्री समाजासाठी हितकारक आहेत असे वाटते का?<br>जर नाही तर मुद्दा क्र ४.६ वर जा.	होय / नाही / माहित नाही
४.५	भटक्या कुत्र्यांचा काय उपायोग आहे असे तुम्हाला वाटते?	पहारा<br>जंगली प्राण्यांना दुर ठेवण्यासाठी<br>चोरांना दुर ठेवण्यासाठी<br>इतर
४.६	भटकी कुत्री उपद्रवी किंवा त्रासदायक आहेत असेतुम्हाला वाटते का?	होय / नाही / माहित नाही
४.७	गावातील भटकी कुत्री माणसाच्या आरोग्यासाठी धोकादायक आहेत का?	होय / नाही / माहित नाही
४.८	या भटक्या कुत्र्यांचे पोट कसे भरत असेल?	गावातील लोकांकडुन<br>कचरा कुंडी<br>रस्त्यावरील कचरा<br>इतर<br>माहीत नाही

४.९	तुम्ही भटक्या कुत्र्यांना खायला घालता का? जर होय तर पुढील प्रश्न सोडवा नाही तर मुद्दा क्र ४.१३ वर जा.	होय / नाही / माहित नाही
४.१0	भटक्या कुत्र्यांना खायला दिल्याने पुण्य मिळते असे तुम्हाला वाटते का?	होय / नाही / माहित नाही
४.११	भटक्या कुत्र्यांना खायला देणे त्यांच्यावर दाखवलेले प्रेम किंवा दया आहे असे तुम्हाला वाटते का?	होय / नाही / माहित नाही
४.१२	शिल्लक जेवण वाया घालवण्यापेक्षा ते भटक्या कुत्र्यांना खायला देणे जास्त चांगले आहे असे तुम्हाला वाटते का?	होय / नाही / माहित नाही
४.१३	तुमच्या भागातील भटक्या कुत्र्यांचे आरोग्य कसे आहे?	चांगले<br>मध्यम<br>वाईट
४.१४	जर तुम्हाला भटका कुत्रा जखमी अवस्थेत दिसला तर तुम्ही त्याला पशुवैदयाकडे घेवुन का?	होय / नाही / माहित नाही
४.१५	जी व्यक्ती अशा कुत्र्यांना खायला घालते व रहायला देते ती व्यक्ती या कुत्र्यांच्या आरोग्याची व लसीकरणाची जबाबदारी घेते का?	होय / नाही / माहित नाही
४.१६	भटक्या कुत्र्यांच्या आरोग्याची जबाबदारी सरकारने घ्यावी का?	होय / नाही / माहित नाही
४.१७	कुत्र्यांच्या संख्येवर नियंत्रण ठेवण्यासाठी मागील दोन वर्षात काही कार्यक्रम झाल्याचे तुम्हाला माहीत आहे का?	होय / नाही / माहित नाही
४.१८	पुढीलपैकी कोणत्या मार्ग भटक्या कुत्र्यांच्या संख्यवर नियंत्रण ठेवु शकतो?	मारून टाकणे<br>कोंडवाडयात टाकणे<br>प्राजनन व्यवस्थापण<br>कचऱ्याचे चांगले व्यवस्थापण<br>इतर<br>माहीत नाही

५. कुत्र्यांच्या संख्येवीषयी (फक्त पाळीव कुत्र्यांच्या मालकांसाठी)

५.१	तुमच्याकडे किती कुत्रे आहेत?	कुत्रा<br>कुत्री<br>एकुण
५.२	त्यांचे वय कीती आहे?	
५.३	कुत्र्यांची जात कोणती?	
५.४	तुमचा कुत्रा तुम्हाला कसा मिळाला?	विकत घेतला<br>भेट मिळाली<br>दत्तक घेतला<br>स्वतःच्या कुत्रीचे पिल्लु<br>इतर
५.५	गावठी कुत्र्यांपेक्षा जातीवंत कुत्रे पाळणे तुम्हाला जास्त आवडते का? होय असल्यास पुढील प्रश्न सोडवा नाही तर मुद्दा क्र ५.७ वर जा.	होय / नाही / माहित नाही
५.६	जातीवंत कुत्रे गावठी कुत्र्यांपेक्षा चांगले असतात असे तुम्हाला का वाटते?	लोकांमधील पत<br>कुत्र्यांची बुदिध्मत्ता<br>स्वच्छता<br>इतर<br>काही ठरावीक कारण नाही
५.७	तुमचा कुत्रा तुमच्या घराच्या आवारात राहतो का? होय असल्यास पुढील प्रश्न सोडवा नाही तर मुद्दा क्र ५.९ वर जा.	होय / नाही
५.८	घराबाहेर जाताना कुत्रा तुमच्या किंवा तुमच्या घराच्यांसोबत जातो का?	नेहमी जातो<br>कधीतरी<br>क्वचीत<br>कधीच नाही
५.९	तुमचा पाळीव प्राणी नोंदणीकृत आहे का?	होय / नाही / माहित नाही
५.१०	मागील वर्षी तुमच्या कुत्र्याला लसीकरण केले आहे काय? जर नाही तर मुद्दा क्र ५.१३ वर जा.	होय / नाही

५.११	मागील वर्षी तुमच्या कुत्र्याला कीती वेळा लसीकरण केले आहे?	
५.१२	तुमच्या कुत्र्याला रेबीजची लस दिली आहे का?	होय / नाही / माहित नाही
५.१३	जर दिली असेत तर लस कधी दिली गेली आहे?	
५.१४	तुमच्या कुत्र्याची प्रजनन न होण्यासाठीची शस्त्रक्रिया केली आहे का?	होय / नाही / माहित नाही
५.१५	तुमच्या कुत्र्याची शस्त्रक्रिया का केली नाही?	होणारा खर्च<br>सेवा उपलब्ध नाही<br>माहिती नाही<br>प्रजनन रोखण्याचे काम क्रुर वाटते<br>इतर<br>काही कारण नाही

Consent form for the survey of community members

Project Title: A survey to determine the knowledge, attitudes and practices of people on dogs and dog-related rabies.

Harish Kumar Tiwari, a PhD student at Murdoch University is conducting a survey to determine the knowledge, attitudes and behavioural practices of people from different communities towards the dog population and its role in the spread of rabies. The study is designed to identify factors that may influence the occurrence of rabies in India and the level of awareness about the disease. This study is being done in collaboration with the School of Veterinary and Life Sciences, Murdoch University, Perth, Australia and Ashoka Trust for Research on Environment and the Ecology, Bangalore, Karnataka, India.

The questionnaire will take approximately 30 minutes to complete. The first part of the questionnaire covers general household information and the latter part deals with awareness about dogs and rabies. The study will help evaluate the existing strategies to control rabies and the dog population in India.

Your participation in this survey is greatly appreciated. All information collected from you will be kept strictly confidential and no information that may identify you will be used in any report or publication. You may withdraw from the questionnaire at any stage if you wish without any impact on you.

Consent:

Do you have any questions about this study?	Yes	No
Do you understand the purpose of the study and your involvement?	Yes	No
Would you like to participate in the study?	Yes	No

Please sign to record your consent to participate in this study ____________________

OR

This consent form with the requirements and conditions associated with this questionnaire survey has been read out to me in the language I understand and I consent to participate in this survey.

____ (Left Thumb Impression)

If you have any queries or concerns regarding this survey you can contact the Human ethics office at Murdoch University or email at Human.Ethics@murdoch.edu.au.

Thank you for your assistance with this project.

Sincerely

Harish Kumar Tiwari
PhD Student
School of Veterinary and Life Sciences
Murdoch University
South Street, Murdoch 6150
Western Australia
Phone : 0426842710
Email: h.tiwari@murdoch.edu.au

<u>समुदायाच्या सदस्यांच्या सर्वेक्षणासाठी मंजूरी फॉर्म</u>

प्रकल्प शीर्षक: कुत्रे आणि कुत्रा-संबंधित रेबीजवरील लोकांच्या ज्ञानाचे, वागण्याचा आणि अभ्यासांचे निर्धारण करण्यासाठी एक सर्वेक्षण.

मर्डोक विद्यापीठातील पीएचडी विद्यार्थी हरीश कुमार तिवारी, वेगवेगळ्या समुदायातील लोकांच्या कुटूंब्यासाठी आणि रेबीजच्या प्रसारणात त्यांची भूमिका यासाठी ज्ञान, दृष्टीकोन आणि वर्तनात्मक पद्धती निर्धारित करण्यासाठी सर्वेक्षण करीत आहेत. भारतातील रेबीजच्या परिणामावर आणि रोगाविषयी जागरूकता पातळीवर प्रभाव पाडणारी कारणे ओळखण्यासाठी हा अभ्यास तयार करण्यात आला आहे. हा अभ्यास पशुवैद्यकीय आणि जीवन विज्ञान शाळेच्या शाळा, मर्डोक विद्यापीठ, पर्थ, ऑस्ट्रेलिया आणि अशोक ट्रस्ट फॉर रिसर्च ऑन एनव्हायर्नमेंट अँड इकोलॉजी, बँगलोर, कर्नाटक, भारत यांच्या सहकार्याने करीत आहे.

प्रश्नावली पूर्ण होण्यास सुमारे 30 मिनिटे लागतील. प्रश्नावलीतील पहिल्या भागात सामान्य घरगुती माहिती समाविष्ट असते आणि त्यानंतरचा भाग कुत्रे आणि रेबीजविषयी जागरूकता हाताळतो. भारतातील रेबीज आणि कुत्रांची संख्या नियंत्रित करण्याच्या विद्यमान धोरणांचे मूल्यांकन करण्यात मदत होईल.

या सर्वेक्षणात आपले सहभाग मोठ्या प्रमाणात कौतुक केले जाते. आपल्याकडून गोळा केलेली सर्व माहिती कठोरपणे गोपनीय ठेवली जाईल आणि आपल्याला ओळखणारी कोणतीही माहिती कोणत्याही अहवालात किंवा प्रकाशनामध्ये वापरली जाणार नाही. आपण आपल्यास कोणताही प्रभाव न घेता, आपण कोणत्याही प्रश्नावरून प्रश्नावलीतून मागे जाऊ शकता.

संमती:

आपल्याकडे या अभ्यासाबद्दल काही प्रश्न आहेत का?	होय	नाही
अभ्यासाचा हेतू आणि आपल्या गुंतवणूकीचा उद्देश समजला आहे का?	होय	नाही
आपण अभ्यासात सहभागी होऊ इच्छिता?	होय	नाही

कृपया या अभ्यासात सहभागी होण्यासाठी आपली संमती नोंदविण्यासाठी साइन इन करा

किंवा

या प्रश्नावली सर्वेक्षणाशी संबंधित असलेल्या अटी आणि शर्तींशी हा संमती फॉर्म मला समजलेल्या भाषेत वाचला गेला आहे आणि मी या सर्वेक्षणात सहभागी होण्यास सहमती देतो.

__________________________ __________________________

(स्वाक्षरी) (डावा थंब इंप्रेसियन)

या सर्वेक्षणासंबंधी आपणास काही शंका किंवा शंका असल्यास आपण मर्डोक विद्यापीठातील संपर्क साधू शकता किंवा Human.Ethics@murdoch.edu.au वर ईमेल करु शकता.

या प्रकल्पाच्या सहाय्याने धन्यवाद.

हरीश कुमार तिवारी
पीएचडी विद्यार्थी
मर्डोक विद्यापीठातील
पशुवैद्यकीय व जीवन विज्ञान शाळेचे शाळा
साउथ स्ट्रीट, मर्डोक 6150
वेस्टर्न ऑस्ट्रेलिया
फोन: +61-426842710
ईमेलः h.tiwari@murdoch.edu.au

Harish Kumar Tiwari PhD Student
School of Veterinary and Life Sciences
Murdoch University
South Street,
Murdoch 6150
Western Australia
Phone: +61-426842710
Email: h.tiwari@murdoch.edu.au
